# Supplementary material for: Effect of water-based aerobic training on anthropometric, biochemical, cardiovascular, and explosive strength parameters in young overweight and obese women: a randomized controlled trial
Source: PeerJ. 2025 Feb 21;13:e19020. doi: 10.7717/peerj.19020 (PMC11849503; doi:10.7717/peerj.19020)
Supplement: Supplemental Information 2 [file peerj-13-19020-s002.pdf]

PARTICIPANT INFORMATION SHEET

Effect of water-based aerobic training on anthropometric, biochemical, cardiovascular, and explosive strength parameters in young overweight and obese women

Dear Participant,

Thank you for showing an interest in our study. Here is some further information regarding the nature of our investigation, along with the answers to some questions you may be wishing to ask. A memberof our research team will go through this information sheet with you and answer any further questions directly. Please do ask if you wish for clarity over any matter.

Location of the study:

The study will take place in the High institute of Sport and Physical Education of Kef, University of Jendouba.

Investigators:

Dr Imen Ben Cheikh

Who is funding the research?

This research study is funded by the local Research Unit: Sport sciences, Health and Movement.

Can I take part in the study?

We are looking for individuals who fulfill the following criteria:

- Female
- Aged 20-35 years old
- Inactive (less than 150 minutes of moderate-intensity aerobic physical activity per week)
- BMI 25-35 kg.m-^{2}
- A stable state of health, specifically not having any infectious or skin diseases
- Experienced with water-based exercise routines
- Willing to strictly comply with all study procedures and restrictions
- Willing to participate, as demonstrated by voluntary written informed consent

Those who meet the following criteria will, unfortunately, be deemed unable to participate:

- Having a history of cardiovascular disease, hypercholesterolemia, diabetes, arterial hypertension, orthopedic limitations, water phobia or any other contraindication for aquatic exercise, and inability to safely enter and exit the pool.
- Currently participating in another clinical trial deemed to potentially interfere with this study
- Current or recent (within the last 30 days) smoker

- Currently taking prescription or non-prescription medication that may interfere with metabolism (including beta-blockers, insulin, bronchodilators, anti-inflammatory agents, thyroxine and medication/supplements that in the opinion of the investigators may affect metabolism).

What is the study about?

Our goal is to optimise physical activity interventions to help previously inactive women improve their body shape and composition. Many people seeking to lose weight or improve body composition initiate an exercise program. Traditional modalities of exercise (i.e., walking, running) are linked to a higher risk of musculoskeletal injury because they put accumulated load on the lower limbs. This risk is specially high in obese individuals, even if the health advantages of aerobic exercise training have been demonstrated. Their overweight leads to significant stress, even pain in the joints of the lower limbs, and seems to be a constraint for carrying out physical activity. However, physical activities with low joint impacts such as swimming, water aerobics, or deep water running are preferred and become more tolerated by overweight or obese persons than land exercises. The available studies show significant variation in participant age, intensities and durations, objectives and program designs, casting doubt on their actual effects and complicating the comparison of results. Therefore, further information is warranted to comprehend the impact of the adequate aquatic programs in genuine scenarios on improving obese women's health. It remains uncertain how a short-term water-based aerobic program using interval training at moderate to high intensities might health risk factors (e.g., weight loss, BMI, biochemical and cardiovascular parameters) and physical fitness in inactive young obese women.

Our research aim is therefore to assess the effects of 10-week water-based aerobic training without nutritional intervention (thrice a week) on anthropometric, biochemical, cardiovascular, and explosive strength of upper and lower limb parameters in young overweight and obese women.

How will I be involved?

Participation in this study will involve completing five preliminary visits (i.e., 3 familiarization sessions and 2 testing sessions) to the High institute of Sport and Physical Education of Kef, followed by the completion of a 10-week training period. The study will conclude with a final visit to the Institute at the end of the intervention.

<u>Preliminary visits 1-3: consent and familiarisation</u>

In the first of your visits to the High institute of Sport and Physical Education of Kef, University of Jendouba, a member of our research team will recap the information within this document and you will be given the opportunity to ask any questions that you may have. If and when you are happy with the details of the study, a consent form will be signed. You will be familiarised with the experimental procedures to ensure your technical proficiency in performing testing and training procedures, and with the Borg Scale of Perceived Exertion 6-20 (RPE) (all briefly described later in this document).

<u>Preliminary visits 4-5: pre-intervention testing</u>

On the 4th visit, you will arrive at the local Research Unit of the High Institute of Sport and Physical Education of Kef between 08:00 and 08:30am following an overnight fast (nothing from 08pm the night before,except water) and after abstaining from exercise the

day before. The first few measurements need to be taken both fasted and rested, so we will ask you to travel by car or public transport and to take the lift inside the building to the research unit, to keep activity to a minimum. Once in the research unit, you will complete a number of tests, in the sequence shown below. The procedure for each is as follows:

- Anthropometric Measures: Your body weight and height will be measured. In addition, triceps, suprailiac and thigh skinfolds will be measured to estimate the body body fat. Before measurements you will be asked to use the least amount of clothing possible.
- Fasting blood sample: If you are willing to donate a blood sample, a 4ml sample will be obtained from the antecubital vein in the arm by a trained and experienced member of our research team.
- Blood pressure and heart rate assessment: We will measure your blood pressure using a hand-held sphygmomanometer and stethoscope, and heart rate will be recorded with a telemetric system. The readings were taken after 10-min rest in a seated position and a noise-free environment.

On the evening of the 5th visit (19:00 – 21:00), you will arrive at the indoor basketball court of the local physical education school between 07:00 and 09:00pm to perform three physical tests (Squat Jump, Counter movement Jump, and Seated Medicine Ball Throw - 3kg). We recommend you wear something comfortable for exercising indoors, such as a T-shirt, shorts and trainers. To ensure that you do not leave us hungry, you will be provided with a lunch meal, consisting of sandwiches and fruit, as well as hot and cold drink options.

10-week intervention

Once baseline data have been completed, you will be allocated to one of two trial conditions: experimental condition (performing a water aerobics training program) or a control condition (maintaining their usual activities for 10 weeks). You will be asked not to make any changes to your daily diet and not to engage in any dietary practices that would result in weight loss.

Experimental Condition: If in this group, you will be instructed by a qualified swimming instructor and under the supervision of an experienced therapist. The intervention will require you to do 50 minutes of Water activities on 3 days of the week (Monday, Wednesday, and Friday from 06:30 to 07:30 pm) for 10 weeks, including warm-up (10 min), main activities (30 min), and cool-down (10 min). In each session, you will start with a warm-up (10 min) including static walking, a combination of stretching exercises, and water walking for a range of motion and relaxation. The main part will adopt an interval training method, consisting of 6 sets of 3-min effort interspersed by 2 min of active interval at RPE 9 (lighter intensity interval). You will perform 10 exercises by combining 4 upper and 3 lower limb movements. The training load progressively increased during the 10 weeks to minimize the risk of the occurrence of possible injuries in this particular musculature. Borg's RPE 6-20 Scale will be used to decide on the intensity of the program and to assess your progression, which was presumed to be maintained between 12 and 13 RPEs in the first mesocycle (somewhat hard) and between 14 and 16 RPEs in the second mesocycle (hard). The last part would be a cool-down activity (i.e., stretching, deep breathing technique, and relaxation and self-care free water activity). The swimming pool where the intervention will take part will be 1.50 m deep with water and air temperatures of 30-32°C and 27-28°C, respectively.

Control condition: If allocated to this condition, you will be asked to maintain your normal activities and diet. You will be added to a waiting list to receive either exercise intervention after completing the10-week trial period, so that you do not miss out on the opportunity to

receive the exercise intervention.You will be able to choose with intervention you wish to follow, with post-intervention measures available, should you wish.

<u>During- and post-intervention testing session:</u>
The procedure of the pre-intervention testing session will be repeated on two days during week 12.

How do I benefit from participation?

By taking part in this study, you will undertake a supported, structured exercise intervention which, if adhered to should improve fitness and health. You will also receive a vast range on physiological, biochemical, and anthropometric information about yourself and your body. Such information is not only of great interest, but could help guide you toward improving aspects of fitness and health in the future.

Are there any risks involved in participation?

The most obvious risks to you will involve the blood sampling.

The insertion of a needle, used to obtain the blood samples can sometimes cause mild discomfort; however all of the researchers involved are trained, skilled phlebotomist, and will do their utmost to prevent this. Once the needle is positioned, you should be largely unaware of its presence and the extraction of blood is a painless process. After the removal of the needle, there is a chance that a small amount of bruising, at the point of insertion, may occur. Application of pressure to this site by the researcher, after removing the needle, will help reduce this risk.

Finally, if at any point during the protocol you feel uncomfortable or unable to continue, testing will beceased immediately.

Can I change my mind?

You can withdraw from the study at any point, without giving reason. If you choose to withdraw you would be asked which type of withdrawal you would prefer – you can choose between leaving the study and allowing the information already given to be used by the study team OR leaving the study and asking for the information already given by you to be destroyed. If you do not wish to participate, you will continue to be treated as before.

What happens to my information?

Research data stored in paper form, such as your age, weight, height, address and medical information will be stored in a locked filling cabinet in the principal researcher's office. You will be assigned a unique study ID and experimental data will be stored using this unique ID number on a password protected computer for analysis purposes. Data will only be accessed by the principal investigator or members of the research team. Blood samples will be stored in the Local Research Unit freezers at the High Institute of Sport and Physical Education. Research data will be retained intact for a period of 10 years from the date of collection.

Should you withdraw from the study, your research data will be destroyed, and subsequently your data will not be included in any publications. The result of the study is expected tobe published in a scientific journal, but your name will never be published. All data will remain completely confidential at all times.

What happens if something goes wrong on the day of a trial?

The High Institute of Sport and Physical Education of Kef holds insurance policies which apply to this study. If you are harmed in any way by taking part in this research project your normal rights apply and you may have grounds for legal action.

Complaints procedure:

If you have any complaints regarding the way you have been treated or anything else relating to the study you can write to Dr Anissa Bouassida who will investigate the matter fully.

Dr Anissa Bouassida
Head of the Research Unit
High Institute of Sport and Physical Education of Kef
Campus Universitaire Boulifa,
El Kef
7100
Tunisia
Tel: +216 98422655
Email: bouassida_anissa@yahoo.fr

What do I do next?

If you feel you would like to be involved in our study, have any questions, or would like some further information, please contact Dr Imen Ben Cheikh so we can arrange a meeting. If you confirm that you do wish to participate, a time and date for the initial visit will be arranged, where you will be asked to complete an informed consent form to confirm that you are happy to participate in this study.

Who has ethically reviewed the project?

This study has been reviewed and approved by the Local Research Ethics Committee of the High Institute of Sport and Physical Education of Kef, University of Jendouba, Tunisia.

Contacts:

Dr Imen Ben Cheikh
Phone: +216 99727857/ +216 53227857
Email: ben_chiekh_imen@yahoo.fr
